# Changes in the Sterol Composition of the Plasma Membrane Affect Membrane Potential, Salt Tolerance and the Activity of Multidrug Resistance Pumps in *Saccharomyces cerevisiae*

**DOI:** 10.1371/journal.pone.0139306

**Published:** 2015-09-29

**Authors:** Marie Kodedová, Hana Sychrová

**Affiliations:** Department of Membrane Transport, Institute of Physiology of the Czech Academy of Sciences, Prague, Czech Republic; Faculty of Pharmacy, University of Lisbon, PORTUGAL

## Abstract

We investigated the impact of the deletions of genes from the final steps in the biosynthesis of ergosterol (*ERG6*, *ERG2*, *ERG3*, *ERG5*, *ERG4*) on the physiological function of the *Saccharomyces cerevisiae* plasma membrane by a combination of biological tests and the diS-C_3_(3) fluorescence assay. Most of the *erg* mutants were more sensitive than the wild type to salt stress or cationic drugs, their susceptibilities were proportional to the hyperpolarization of their plasma membranes. The different sterol composition of the plasma membrane played an important role in the short-term and long-term processes that accompanied the exposure of *erg* strains to a hyperosmotic stress (effect on cell size, pH homeostasis and survival of yeasts), as well as in the resistance of cells to antifungal drugs. The pleiotropic drug-sensitive phenotypes of *erg* strains were, to a large extent, a result of the reduced efficiency of the Pdr5 efflux pump, which was shown to be more sensitive to the sterol content of the plasma membrane than Snq2p. In summary, the *erg4*Δ and *erg6*Δ mutants exhibited the most compromised phenotypes. As Erg6p is not involved in the cholesterol biosynthetic pathway, it may become a target for a new generation of antifungal drugs.

## Introduction

The plasma membrane, together with the cell wall, protects the yeast cell from environmental changes. A proper plasma-membrane composition is essential and has to be carefully controlled by the cell. The regulation of membrane composition has an important modulatory role and serves as an adaptive response to variations in temperature, pH, hydration etc. [1–2]. The regular plasma-membrane barrier function is dependent on its permeability and fluidity, which are determined by various aspects, such as the length and saturation of lipid acyl chains and the amount and type of sterols and sphingolipids in the membrane [3–4]. In yeast cells, sterols not only contribute to the fluidity of lipid membranes, but also ensure many other vital processes including vesicle formation and protein sorting, cytoskeleton organization, endocytosis and mating [2,5–8].

Yeast cells synthesize their major sterol, ergosterol, in the membrane of the endoplasmic reticulum via a cascade of coupled enzymatic reactions. Ergosterol is then transported from the site of its synthesis to the plasma membrane. However, *Saccharomyces cerevisiae* is also a facultative anaerobic organism, which becomes a sterol auxotroph in the absence of oxygen. Under these conditions, cells take up sterols from the environment, incorporate them into the plasma membrane and transport them back into the membrane of the endoplasmic reticulum, where the free sterols become esterified and the resulting steryl esters are stored in lipid droplets [7,9]. Surprisingly, the synthesis of ergosterol in lower eukaryotes requires more energy than the synthesis of mammalian cholesterol [10]. Only some less evolutionary advanced species of fungi, which live in an aquatic medium where the hydration is stable, synthesize cholesterol instead of ergosterol [11]. Under special conditions, mammalian cholesterol can substitute for the crucial role of ergosterol, e.g. some pathogenic fungi, such as *Candida glabrata* and *Aspergillus fumigatus*, import exogenous cholesterol from the host serum in the presence of oxygen to counteract the toxicity of antifungal agents that target ergosterol and its synthetic pathway [12–14]. Both cholesterol and ergosterol increase the mechanical resistance of the plasma membrane to osmotic treatments, but ergosterol and its precursors provide lipids with a better protection against peroxidation than cholesterol [15]. Therefore, the ergosterol biosynthetic pathway might have accompanied the evolution of the fungi kingdom and the properties of sterols were gradually optimized for their function in the biosynthetic pathway [1].

**Fig 1:**
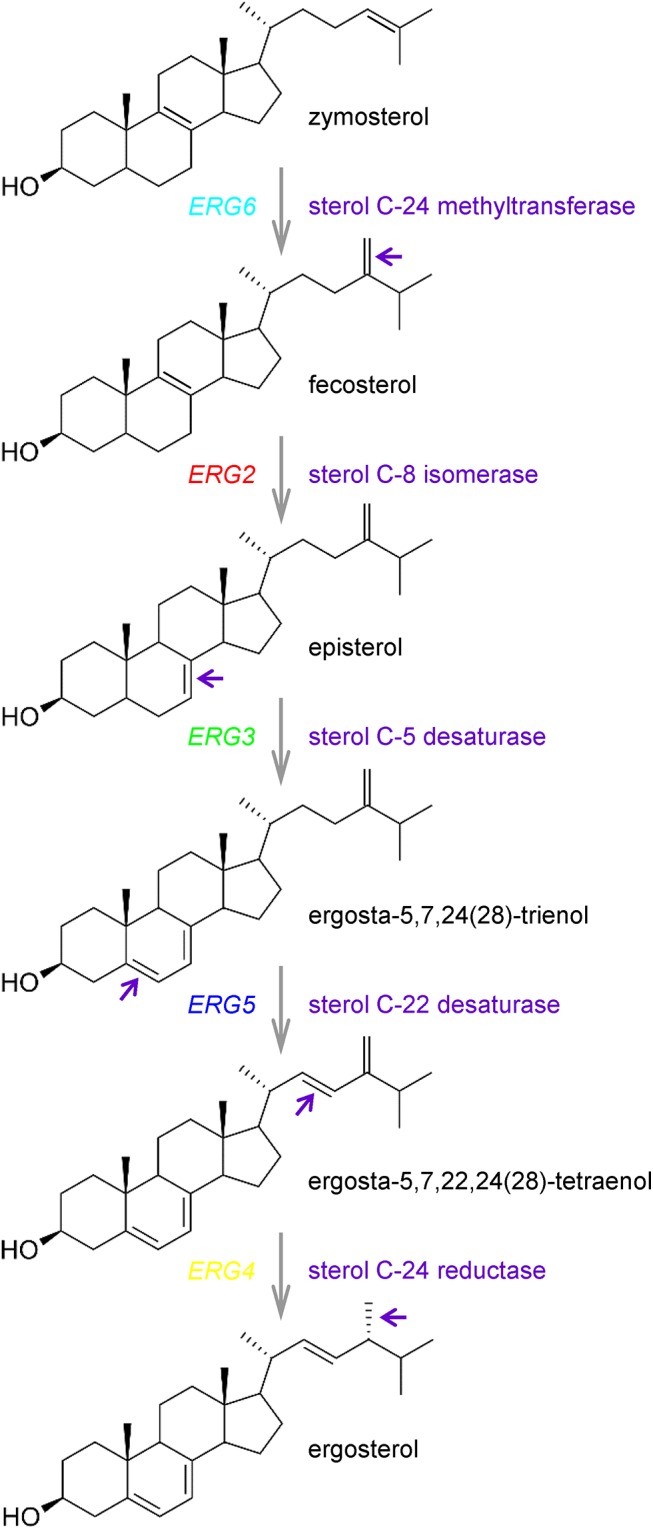
Final steps of ergosterol biosynthesis in *S*. *cerevisiae* with highlighted targets of studied Erg proteins. Violet arrows indicate parts of sterol molecules that were changed by the corresponding enzymes.

The synthesis of ergosterol in yeast cells is a complex process. Altogether the ergosterol biosynthetic pathway involves over 20 distinct reactions [16]. The early pathway starts with acetyl-CoA and ends with the formation of farnesyl pyrophosphate, an important intermediate which is the starting point for several essential pathways. Therefore mutations in this part of the pathway are lethal, because a number of essential metabolic products cannot be synthesized [16]. The next three steps catalyzed by the enzymes encoded by *ERG9* (squalene synthase), *ERG1* (squalene epoxidase) and *ERG7* (lanosterol synthase) are also essential, and they lead to the synthesis of the first sterol molecule, lanosterol [9,17]. Strains with deletions of genes encoding enzymes for the late steps of ergosterol biosynthesis are viable and accumulate sterols that differ from ergosterol in the number and position of double bonds in the B-ring and the side chain of the sterol molecule [17] as shown in Fig 1.

Ergosterol plays a crucial role in the architecture of the yeast plasma membrane. The plasma membrane of *Saccharomyces cerevisiae* is composed of structurally distinct lateral microdomains which are formed through the association of sterols and sphingolipids with proteins. Many membrane proteins are distributed non-homogenously in patterns ranging from discrete ergosterol-enriched MCC patches (**m**embrane **c**ompartment of arginine permease **C**an1p) to nearly continuous MCP networks (**m**embrane **c**ompartment of **P**ma1p) [18]. In addition, dynamic patch-like domains, called MCT (**m**embrane **c**ompartment of **T**ORC2), were described for Tor Complex 2 [19]. The domain structure of the plasma membrane has been not only identified in *S*. *cerevisiae* but also in *Schizosaccharomyces pombe* or pathogenic *Candida albicans* and *Cryptococcus neoformans* [20]. In yeast cells, these microdomains house a number of biologically important proteins involved in Na^+^, K^+^ and pH homeostases, nutrient transport, mating, drug efflux and stress response [20]. Integral plasma membrane proteins with similar functions are often localized to numerous coexisting subdomains that overlap only partially, e.g. Pdr5, Pdr12 and Yor1 proteins (MDR pumps) involved in multidrug resistance as efficient drug exporters [21].

As mentioned above, ergosterol is necessary for the regulation of membrane permeability and fluidity, and for regulating the activity of membrane transporters [16]. Thus, due to its indispensable role in yeast physiology, ergosterol and its biosynthesis became major targets in the development of antifungal drugs over the last sixty-five years [22–24]. Some of the drugs used for the treatment of both systemic and superficial fungal infections specifically block ergosterol synthesis and simultaneously have only a small effect on the host cholesterol synthesis. Allylamines inhibit squalene epoxidase (encoded by *ERG1*), whereas morpholines block two separate steps, C-14 sterol reduction (by the product of *ERG24*) and C-8 isomerization (by the enzyme encoded by *ERG2*). Erg11p, catalyzing the demethylation of lanosterol, is a target of azoles, the most widely used and most numerous group of antifungal drugs [23–24]. Polyenes (nystatin, amphotericin B) selectively interact with the final product of the pathway, ergosterol, and form membrane pores resulting in cell death [22].

The aim of this work was to estimate how changes in sterol composition influence various physiological parameters, growth capacities and tolerance to various stresses in *S*. *cerevisiae* cells, as well as identify a promising candidate among the enzymes involved in ergosterol biosynthesis for the development of new antifungal drugs.

## Materials and Methods

### Yeast strains

The *S*. *cerevisiae* strains used in this study are listed in Table 1. For intracellular pH measurement, we transformed BY4741 and *erg* mutants with the pHl-U plasmid [25], which enables the expression of pHluorin (a modified version of green fluorescent protein sensitive to pH) [26]. We prepared several independent *erg6*Δ strains by deletion of the *ERG6* gene in BY4741 with the *loxP-KanMX-loxP* cassette [27], using the primers ScERG6-KanMX-F and ScERG6-KanMX-R. Successful integration of the cassette and deletion of *ERG6* was verified by diagnostic PCR. All primers used for deletion and verification are listed in S1 Table. Three independent transformants with the correctly integrated cassette were obtained and verified to exhibit the same phenotypes. One representative of these strains was used in subsequent experiments.

**Table 1:** Yeast strains used in this study.

Strain	Genotype	Source
BY4741	*MATa his3*Δ*1 leu2Δ0 met15Δ0 ura3*Δ*0*	EUROSCARF
*erg2*Δ	*MATa his3*Δ*1 leu2Δ0 met15Δ0 ura3*Δ*0 erg2*Δ::*kanMX*	EUROSCARF
*erg3*Δ	*MATa his3*Δ*1 leu2Δ0 met15Δ0 ura3*Δ*0 erg3*Δ::*kanMX*	EUROSCARF
*erg4*Δ	*MATa his3*Δ*1 leu2Δ0 met15Δ0 ura3*Δ*0 erg4*Δ::*kanMX*	EUROSCARF
*erg5*Δ	*MATa his3*Δ*1 leu2Δ0 met15Δ0 ura3*Δ*0 erg5*Δ::*kanMX*	EUROSCARF
*erg6*Δ	*MATa his3*Δ*1 leu2Δ0 met15Δ0 ura3*Δ*0 erg6*Δ::*loxP-kanMX-loxP*	This work
*pdr5*Δ	*MATa his3*Δ*1 leu2Δ0 met15Δ0 ura3*Δ*0 pdr5*Δ::*kanMX*	EUROSCARF
*snq2*Δ	*MATa his3*Δ*1 leu2Δ0 met15Δ0 ura3*Δ*0 snq2*Δ::*kanMX*	EUROSCARF

Multicopy plasmids, YEpPDR5 and pGRUPDR5, were constructed for the production of Pdr5p or Pdr5p fused with green fluorescent protein (GFP) at the C-terminus under the control of a constitutive *NHA1* promoter. The plasmids YEpPDR5 and pGRUPDR5 are derivatives of YEp352 [28] and pGRU1 [29], respectively. The fragments containing *PDR5* were amplified from the genomic DNA of *S*. *cerevisiae* BY4741 by PCR with the primers listed in S1 Table. The PCR products were inserted into pNHA1-985 [30] (for YEpPDR5) and pNHA1-985GFP [30] (for pGRUPDR5) using homologous recombination, correct integration of *PDR5* was verified by diagnostic PCR.

### Media and growth assays

Cells were regularly grown at 30°C on YPD (1% yeast extract, 2% peptone, 2% glucose, 2% agar for solid media) or YNB (0.67% yeast nitrogen base without amino acids, 2% glucose, 2% agar for solid media) media supplemented with Brent Supplement Mix (BSM) after autoclaving. For measurements of intracellular pH, in order to diminish the background fluorescence, cells were cultivated in YNB-pH medium (0.17% yeast nitrogen base without riboflavin and folic acid (MP Biomedicals), 0.4% ammonium sulphate, 2% glucose) supplemented with BSM without uracil. For the selection of *S*. *cerevisiae erg6*Δ::*loxP-kanMX-loxP* transformants, G418 was added to YPD medium at a final concentration of 900 μg/mL.

Growth phenotypes of the strains in the presence of salts and drugs were tested both on solid and in liquid media. For testing lithium tolerance, cells growing overnight in liquid media were diluted to OD_600_ = 0.2 in YPD with 100 mM LiCl or without LiCl and cultivated at 30°C. The OD_600_ was measured at 1 h intervals. The plotted values of relative growth after 22 h cultivation are the means ± SD of three independent experiments. To compare the resistance of strains to antifungal drugs, the growth in liquid media was monitored in a 96-well plate reader Elx808 (BioTek) for 24 h at 30°C. 100 μL of YPD media in a well was inoculated with 2 μL of cell suspension OD_600_ = 1. The OD_595_ was measured at 1 h intervals. Growth curves were obtained in duplicates in a broad range of drug concentrations and repeated twice, representative results are shown. The plotted values of relative growth after 16 h cultivation are the means ± SD.

Drop tests were performed with fresh cells of each tested strain resuspended in sterile distilled water and adjusted to the same initial OD_600_ = 0.6. Tenfold serial dilutions were prepared, and 3 μL aliquots of each dilution were spotted on appropriate YPD or YNB agar plates supplemented as indicated in the text. Plates were incubated at 30°C for several days and photographed daily. Each drop test was performed in two parallels and repeated at least three times, representative results are shown.

Disc diffusion tests were performed as previously described [31] for estimating 1) the toxicity of antifungal drugs and 2) the activity of MDR pumps in *erg* mutants. Yeast cells grown to the exponential phase in liquid YPD medium were washed twice with distilled water and resuspended in 10 mM citrate-phosphate buffer (pH 6.0). They were then diluted into top agar (1% yeast extract, 2% peptone, 2% glucose, 1% agar) to OD_600_ = 0.2 and poured onto YPG plates (1% yeast extract, 2% peptone, 2% glycerol, 2% agar). Drug solutions (2 μL) at the concentrations indicated in the text were spotted onto paper discs laid on top of the agar. The plates were photographed after 2 days at 30°C and the diameters of the growth inhibition zones were measured. To determine the activity of the Pdr5 and Snq2 MDR pumps in *erg* strains, fluconazole was added to the discs together with the specific substrates of the pumps (FK506 for Pdr5p, NQO (4-nitroquinoline *N-*oxide) for Snq2p). Representative results of three independent experiments are shown.

### Measurement of relative membrane potential (diS-C_3_(3) assay)

The relative membrane potential of yeast cells was estimated by a fluorescence assay based on the potential-dependent redistribution of the fluorescence probe diS-C_3_(3) (3,3’-dipropylthiacarbocyanine iodide) [32–33], as described in [31]. Cells from the early exponential growth phase were harvested, washed twice with distilled water and resuspended in an assay buffer to OD_600_ = 0.2 and the probe was added to a final concentration of 2 × 10^−8^ M. Fluorescence emission spectra (λ_ex_ = 531 nm, λ_em_ = 560–590 nm) of the cell suspensions were measured in an ISS PC1 spectrofluorimeter equipped with a xenon lamp. The staining curves recorded the dependence of the fluorescence emission maximum wavelength λ_max_ at the time of staining. Representative results of three independent experiments are shown.

### Cell size measurements

Cell diameter was estimated for cells growing in YPD to the early exponential growth phase. The cell size was measured before and 20 s after the addition of NaCl (final concentration 1 M) to the sample. A cell counter (CASY model TT; Roche Innovatis AG) with a 60 μm capillary was used. The experiment was repeated four times, each time 3–5 × 10^4^ cells were analyzed for each strain and each set of conditions. Intervals containing the most typical 60% of the cell population were visualized using a box plot diagram with the mean diameter from the observed interval (2.5–9 μm) inside the box. Average results are shown ± SD.

### Measurement of intracellular pH

Intracellular pH (pH_in_) was estimated using the pH-sensitive green fluorescent protein ratiometric pHluorin as described previously [25–26,34]. Briefly, cultures expressing cytosolic pHluorin were excited with 400/30 or 485/20 nm light, and the emission was registered at 516/20 nm in a Synergy HT reader (BioTek). In all experiments, the background fluorescence of a wild-type culture not expressing pHluorin was subtracted from both signals independently, before the ratio of the two signals was determined. The pH_in_ was measured 20 min after the addition of NaCl (1 M final concentration) or a corresponding volume of water (as a control) to cell suspensions. The pH_in_ signal was calibrated using washed cells permeabilized with digitonin (300 μg/mL) in phosphate-buffered saline (PBS) for 10 min. Cells were collected by centrifugation, washed with PBS and resuspended to an OD_600_ of 0.5 in citrate-phosphate buffers with pH values ranging from 5.6 to 7.6 in 96-well microtitre plates. The emission ratios at 516 nm upon excitation at 400 and 485 nm were determined after 30 min of incubation of permeabilized cells in calibration buffers and plotted against the corresponding buffer pH. All pH_in_ measurements were repeated three times (7–16 replicates in each experiment), pH_in_ values are represented as means ± SD.

### Fluorescence microscopy

Yeast strains with pGRUPDR5 plasmid were grown to the exponential growth phase in YNB medium supplemented with BSM without uracil and observed with a fluorescence microscope (Olympus AX70).

### Statistical analysis

The statistical analyses were performed with SigmaPlot 13. Differences caused by salt stress or antifungal drugs were statistically described using a paired t-test, i.e. a comparison of the values before and after the addition of salt to each strain, or by ANOVA and subsequent *post hoc* test for finding significant differences among yeast strains.

## Results and Discussion

### Changes in the sterol composition of the plasma membrane affect growth phenotypes and membrane potential

We studied the impact of deletions of genes from the final steps in the biosynthesis of ergosterol (*ERG6*, *ERG2*, *ERG3*, *ERG5*, *ERG4*) on the physiological function of the *Saccharomyces cerevisiae* plasma membrane. For our study, we used EUROSCARF strains from the *KanMX* deletion library constructed in the BY4741 background and prepared the *erg6*Δ mutant. Three independent *erg6*Δ candidates were verified to have the same growth phenotypes. Further, one representative of these mutants was used. This *erg6*Δ strain exhibited the slowest growth compared to the other tested *erg* strains (S1 Fig), and indicated that the product of *ERG6* represents a weak spot in ergosterol biosynthesis.

**Fig 2:**
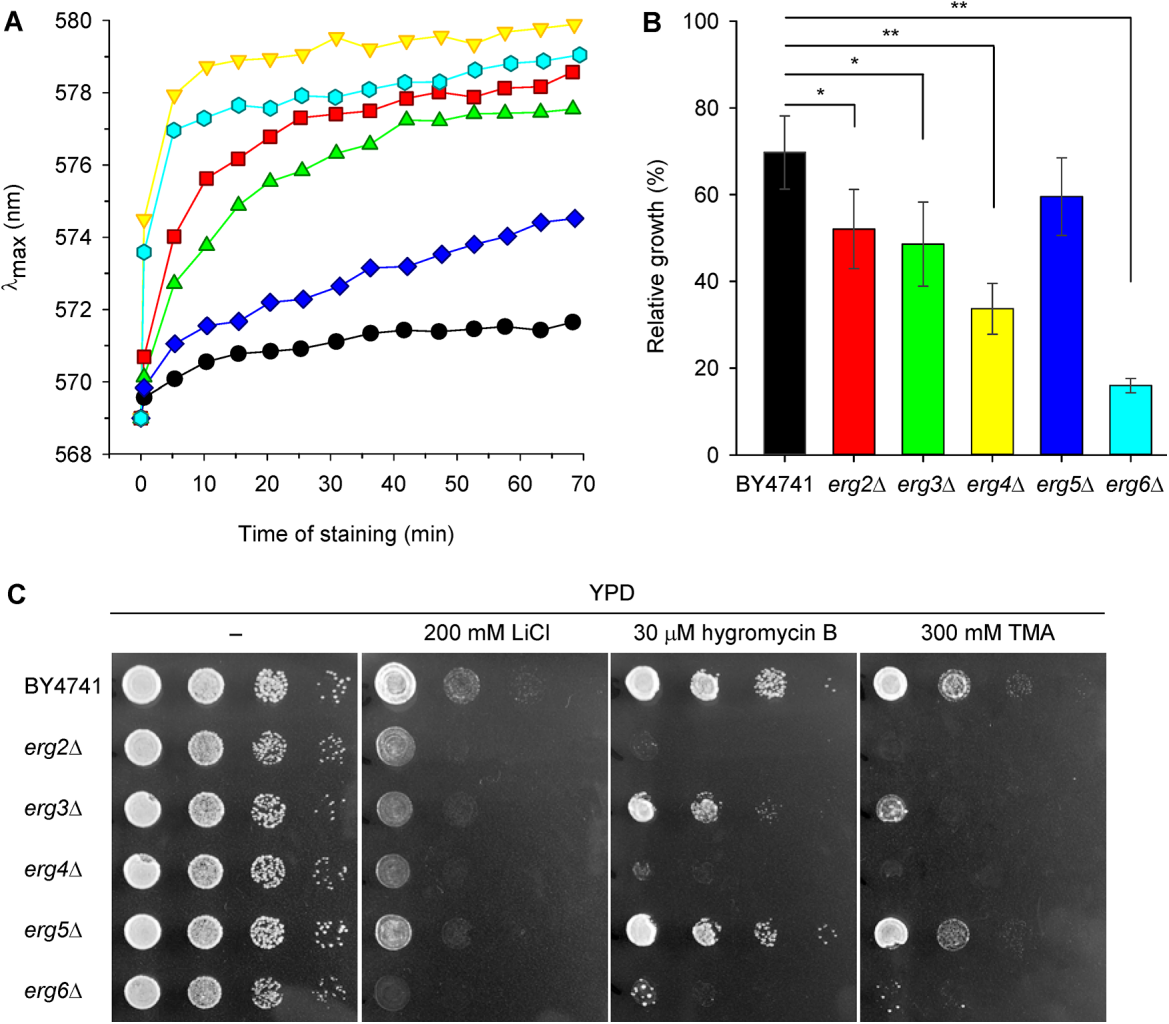
Strains with hyperpolarized membranes are less tolerant to LiCl and cationic drugs. (A) Relative membrane potential of BY4741 (black circles), *erg2Δ* (red squares), *erg3Δ* (green triangles), *erg4Δ* (yellow triangles), *erg5Δ* (blue diamonds) and *erg6Δ* (cyan hexagons) cells estimated with diS-C_3_(3) fluorescence probe. (B) Relative growth in liquid YPD medium supplemented with 100 mM LiCl (growth in YPD medium without LiCl = 100%). The *P* values (*P < 0.05, **P < 0.001) denote statistically significant differences from the wild-type strain. (C) Growth on solid YPD media supplemented with LiCl, hygromycin B and tetramethylammonium (TMA) after 1 day of incubation.

One of the first indicators of plasma membrane damage, due to changes in the properties or lipid and protein content of the plasma membrane, is the inability to maintain plasma-membrane potential. To elucidate whether the accumulation of sterols differing from ergosterol in the plasma membrane of *erg* mutants affects the level of membrane potential, we used a diS-C_3_(3) assay [33]. This assay is based on a cationic potentiometric probe, which is able to sensitively reflect very fine changes in the plasma-membrane potential, such as those caused by membrane lateral microdomain structures [35] or by the altered transport activity of mutated versions of the Trk1 potassium uptake system [36]. According to the diS-C_3_(3) staining of cells, the *erg* mutants divided into three groups (Fig 2A). While the *erg5*Δ strain exhibited almost wild-type level of relative membrane potential (only with a very slight hyperpolarization compared to the parental strain); deletion of the other four genes, and mainly *ERG6* or *ERG4*, had a crucial effect on plasma membrane integrity which resulted in a very rapid probe uptake. They were strongly hyperpolarized, the staining curves had steep inclinations and the equilibrium intracellular concentrations of diS-C_3_(3) were achieved within 10 minutes of staining (Fig 2A). The *erg3*Δ and *erg2*Δ mutants exhibited a less dramatic impairment of membrane function accompanied by a lower hyperpolarization, the influx of the probe into these strains was slower, and the final λ_max_ equilibrium was lower than in *erg4*Δ and *erg6*Δ cells.

It is important to note that the fluorescence response of diS-C_3_(3) to membrane potential is influenced by the activity of the Pdr5 and Snq2 MDR pumps, which actively expel the probe from the cells [37]. It is well known that changes in the composition of the plasma membrane negatively affect the function of many transporters [16,18,38], and thus the observed differences in staining curves (Fig 2A) might be partly due to the different activity of MDR pumps. To verify whether the observed differences in staining reflect purely the hyperpolarization of the membrane, or a decreased activity of MDR pumps, or a combination of both, we performed a series of experiments. First, we complemented the fluorescence measurements of membrane potential with growth assays with lithium chloride in liquid and solid media (Fig 2B and 2C). Toxic lithium cations enter into the cells non-specifically according to the plasma-membrane potential and are actively eliminated from the cytosol by two types of plasma-membrane exporters, Ena1-5 ATPases and the Nha1 cation/H^+^ antiporter, or by the intracellular Nhx1 cation/H^+^ antiporter which sequestrates them into the vacuoles [39]. Differences in the relative growth of wild type and *erg* mutant strains in liquid YPD medium supplemented with 100 mM LiCl (Fig 2B) were in good agreement with the results obtained by diS-C_3_(3) assay, and confirmed the hyperpolarization of the *erg2*Δ, *erg3*Δ, *erg4*Δ and *erg6*Δ plasma membranes. The strain lacking *ERG6* exhibited the poorest growth in the presence of lithium of all the *erg* mutants. This hypersensitivity to Li^+^ has been observed previously [40]. The lithium hypersensitivity of *erg6*Δ mutants was attributed to increased rates of cation influx and not decreased rates of efflux [40], which also corresponds to the hyperpolarization of the cell plasma membrane. Our results, indicating hyperpolarization of the membranes of at least four *erg* mutants, were further verified on solid media with LiCl and two cationic drugs, hygromycin B and tetramethylammonium (TMA). It is presumed that an abnormal sensitivity to these toxic cations does not reflect the activity of MDR pumps, but only a change in the plasma-membrane potential of cells [41]. We observed a similar-looking pattern of yeast sensitivity to the presence of lithium and cationic drugs in the growth media (Fig 2C). The wild-type and *erg5*Δ strains were the most resistant strains, due to their normal or only slightly increased membrane potential, whereas the *erg6*Δ, *erg4*Δ, *erg2*Δ and *erg3*Δ strains were sensitive to all three tested compounds in proportion to the level of hyperpolarization of their membranes.

### *Erg* mutants differ in their tolerance to hyperosmotic stress caused by NaCl and in their intracellular pH

**Fig 3:**
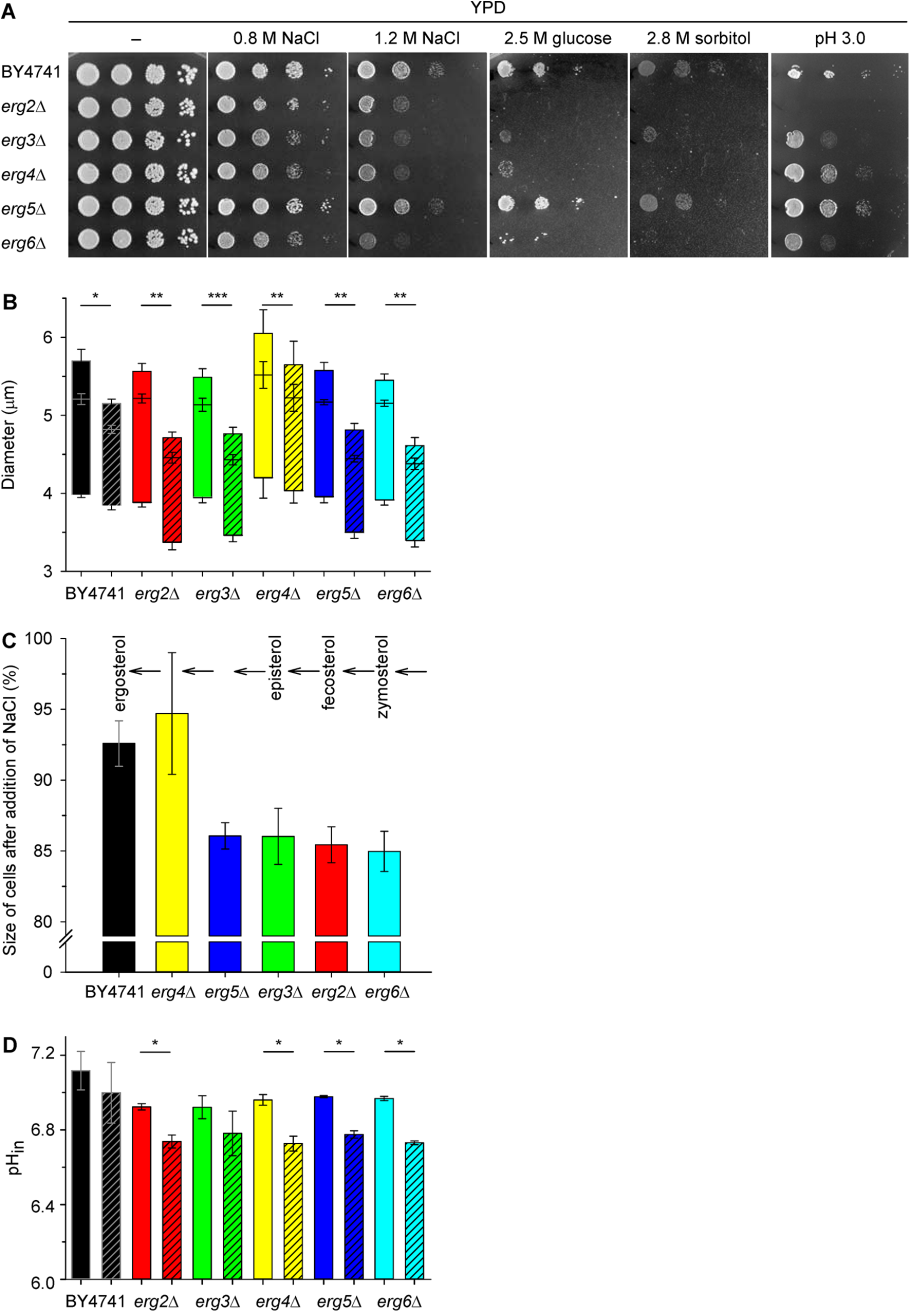
Effect of osmotic stress. (A) Growth on solid YPD medium supplemented with NaCl (recorded after 2 days), with glucose and sorbitol (recorded after 6 days), or adjusted to low pH with tartaric acid (recorded after 1 day). (B) Cell size before (empty boxes) and 20 s after the addition of 1 M NaCl (hatched boxes). The *P* values (*P < 0.01, **P < 0.001, ***P < 0.0001 based on paired t-test) reflect a statistically significant change in cell size after osmotic shock. (C) The shrinkage of cells after osmotic shock caused by NaCl in % (initial size of cells before exposure to 1 M NaCl = 100%). (D) Intracellular pH of cells expressing pHluorin 20 min after the addition of 33 μL of H_2_O (control, empty bars) or 33 μL of 4 M NaCl (final concentration 1 M, hatched bars). The *P* values (*P < 0.05 based on paired t-test) reflect a statistically significant drop in pH after the addition of NaCl.

To further characterize the effects of *erg* mutations, we estimated two other physiological parameters which might be affected by altered plasma-membrane composition, intracellular pH and cell tolerance to hyperosmotic stress. As shown in Fig 3A, wild-type BY4741 strain, together with the *erg5*Δ, tolerated a high external concentration of NaCl much better than the other *erg* mutants. The exposure of yeast cells to saline stress implied exposure to both specific cation toxicity and osmotic stress. Sodium, similarly to lithium, enters cells in a non-specific manner and is actively exported via Ena ATPases and the Nha1 cation/H^+^ antiporter [39]. The intracellular accumulation of sodium in high concentrations is lethal because of its ability to replace necessary potassium cations and to inhibit specific metabolic pathways [39,42]. Lower resistance of the *erg2*Δ, *erg3*Δ, *erg4*Δ and *erg6*Δ strains to hyperosmotic stress is independently demonstrated by their inability to grow in the presence of high concentrations of glucose and sorbitol (Fig 3A). High extracellular osmotic pressure is accompanied by a transient loss of intracellular water visible as cell shrinkage. Fig 3B and 3C show that the addition of 1 M NaCl to cell suspensions diminished the cell size of all strains. The shrinkage of mutants from the earlier steps of ergosterol biosynthesis was greater than the shrinkage of *erg4*Δ and wild-type strains; Fig 3C), which suggested that the native elasticity/rigidity of the membranes of these mutants was severely disturbed. Almost the same level of shrinkage of BY4741 and *erg4*Δ cells (Fig 3C) was surprising, due to the great difference in their tolerance to NaCl in drop tests (Fig 3A). The sensitivity of *erg4*Δ cells to sodium cations is markedly connected to their higher (inside negative) relative membrane potential (Fig 2A) which represents a driving force for sodium influx. On the other hand, the ergosterol precursor accumulated in the membranes of *erg4*Δ cells probably conferred them with a similar elasticity/rigidity to that provided by ergosterol in the wild-type cells (Fig 3C). This result is in agreement with the hypothesis that, during their evolution, the structure of sterol molecules was progressively improved in order to increase their resistance to environmental pressures [1]. It also is worth noting that only the deletion of *ERG4* resulted in a significant increase (*P <* 0.05) in cell size under non-stressed conditions (Fig 3B, empty boxes). YPD-grown cells of all the other mutants had a size similar to those of BY4741. This result is probably related to the earlier observation that *ERG4* is required for proper cell morphology, especially shape remodelling and cell fusion during yeast mating [5,8]. Taken together, the results obtained with high concentrations of NaCl, glucose and sorbitol showed, that it is not only the tolerance to toxic sodium cations that differs among the *erg* mutants, but also their responses to increased osmotic pressure vary according to the sterols present in their membranes.

As we assumed that changes in the membrane’s physical properties (mainly fluidity and potential) affect the activity of plasma-membrane transporters, and as a result the uptake of nutrients and thus the overall cell metabolism, we measured the intracellular pH with the use of pHluorin expression. We observed a significantly lower (*P <* 0.05) intracellular pH in all *erg* mutants exponentially growing in YNB-pH (Fig 3D, empty bars). Further, we tested whether the observed levels of salt tolerance in the mutants and the wild type correlated with the ability to maintain intracellular pH. Indeed, *erg* mutants had problems maintaining pH_in_ homeostasis upon NaCl stress, they exhibited a considerable drop in pH_in_, bigger than in the wild-type strain after 20 min treatment with 1 M NaCl (Fig 3D, compare empty and hatched bars). The highest acidification of the cytosol occurred in the strains with the most hyperpolarized plasma membranes, *erg6*Δ (ΔpH_in_ = 0.24 ± 0.01) and *erg4*Δ (ΔpH_in_ = 0.23 ± 0.05). Hyperpolarization was the reason for the higher sensitivity of the *erg4*Δ and *erg6*Δ cells to toxic cations (Figs 2 and 3A), and it also resulted in a deeper drop in pH_in_ upon NaCl stress. The observed acidification of the cytosol upon salt stress is most probably connected to plasma-membrane proteome changes. Upon the addition of 1 M NaCl, a significant decrease in the abundance of 24 proteins in the *S*. *cerevisiae* plasma membrane was observed, and the Pma1 H^+^-ATPase, which is the main system pumping protons out of cells, was among them [42]. Decreased levels of Pma1p activity in the plasma membrane after salt stress, documented by the acidification of the cytosol (Fig 3D), could be favourable for wild-type cells exposed to toxic sodium ions. A decrease in the activity [31] or amount [42] of Pma1p results in a reduction in the membrane potential (relative depolarization) and a concomitant decrease in the potential-driven influx of toxic sodium cations. On the other hand, the results obtained with *erg* mutants suggest that the above decrease in Pma1p activity at the plasma membrane is not enough to counteract the hyperpolarization brought about by the changes in sterol composition and hence membrane properties. Moreover, *erg* mutants differ not only in their ability to maintain pH_in_ homeostasis upon NaCl stress, but also in the sensitivity to low extracellular pH (Fig 3A). The most sensitive to acidic stress were the *erg2*Δ, *erg3*Δ and *erg6*Δ mutants, whereas the *erg5*Δ strain grew similarly as the wild type.

### *Erg* mutants differ in their susceptibility to antifungal agents

**Fig 4:**
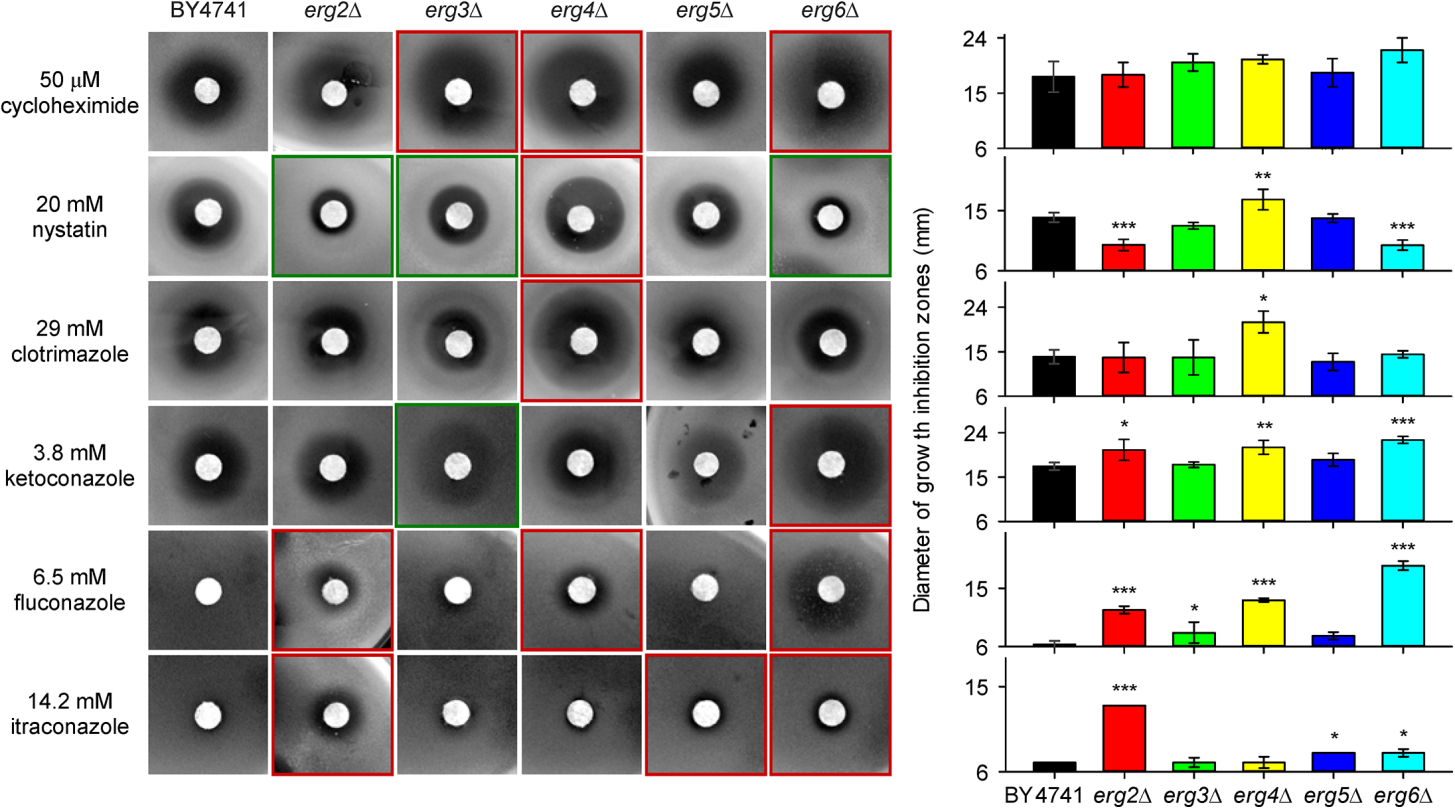
Growth inhibition zones of cells exposed to antifungal drugs. Red frames indicate strains less tolerant to drugs than the wild type, green frames indicate more tolerant strains than the wild type. Diameter of paper discs was 6 mm. The *P* values (*P < 0.05, **P < 0.01, ***P < 0.001) denote statistically significant differences from the wild-type strain.

In comparison to the parental BY4741 strain, most of the *erg* mutants were more sensitive to salt stress or cationic drugs and the level of their sensitivity was connected to the relative hyperpolarization of their plasma membrane (Fig 2 and Fig 3A). We also tested the susceptibility of *erg* mutants to six antifungal drugs with different structures and mechanisms of action (Fig 4 and S2 Fig). Cycloheximide inhibits protein synthesis due to its binding to eukaryotic ribosomes [43], nystatin binds to ergosterol within the cell membrane to generate pores, causing cell membrane leakage and loss of cytoplasmic content [22,44], and imidazoles (clotrimazole, ketoconazole) and triazoles (fluconazole, itraconazole) act as inhibitors of ergosterol synthesis [24]. Disc diffusion tests showed that the *erg6*Δ, *erg4*Δ and *erg3*Δ strains were more sensitive to cycloheximide than the wild type (Fig 4). The mutant lacking *ERG4* was also slightly more sensitive to nystatin than the parental strain, but the deletion of *ERG2*, *ERG6* and to a lesser extent *ERG3* conferred a resistance to this polyene, probably due to a lower affinity of this drug for fecosterol, zymosterol and episterol, respectively, which are accumulated in these mutants. The most susceptible strains to azoles were *erg6*Δ, *erg4*Δ and *erg2*Δ (Fig 4). The heterogeneous sensitivity of the tested *erg* mutants was probably not only influenced by the higher permeability of their membranes for these drugs, but it also suggested a distinct functioning of their MDR pumps.

### Changes in the sterol composition of the plasma membrane affect the activity of Pdr5p

**Fig 5:**
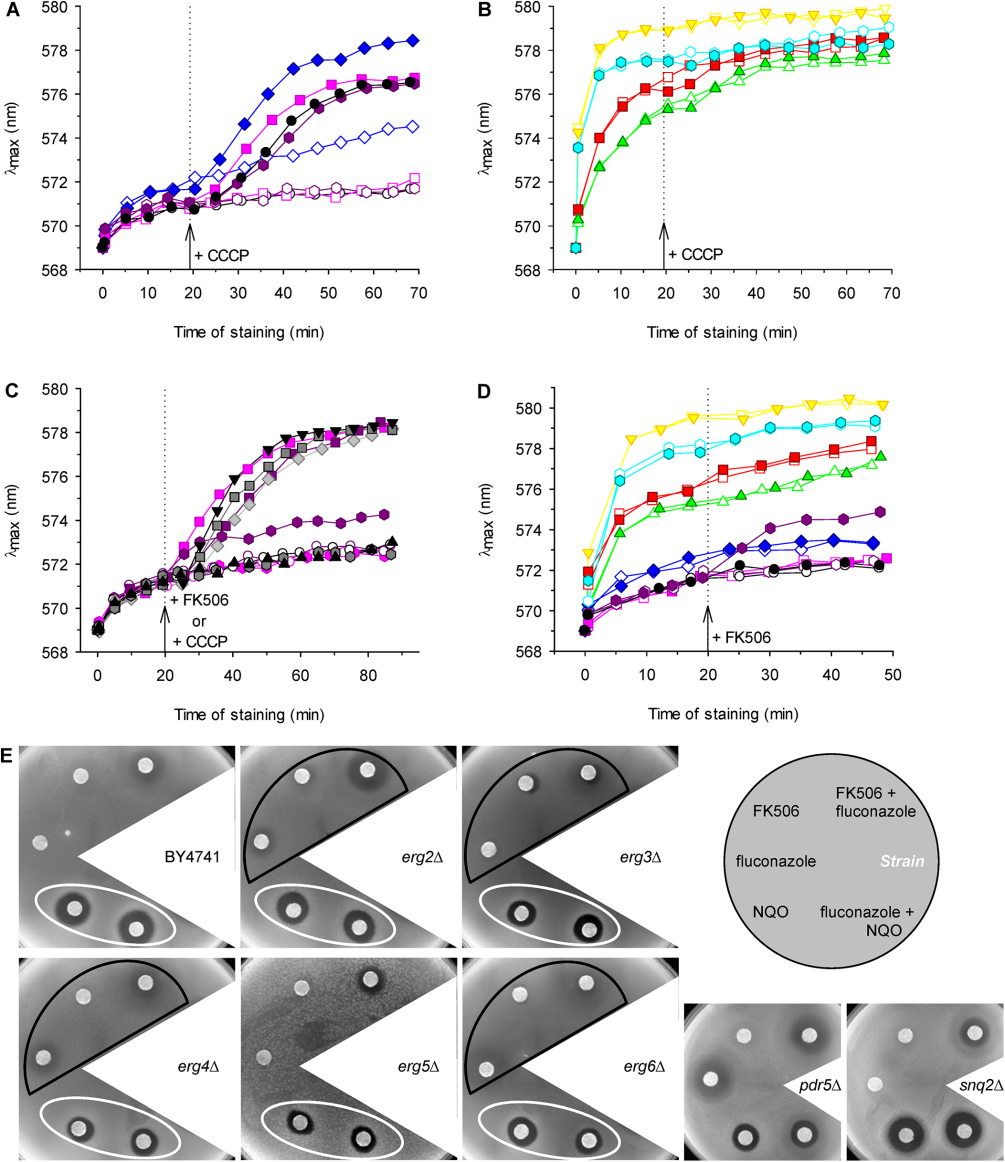
Activity of Pdr5 and Snq2 pumps. Changes in diS-C_3_(3) staining of (A) wild-type BY4741 (black circles), *erg5*Δ (blue diamonds), *pdr5*Δ (pink squares), *snq2*Δ (violet hexagons) and (B) *erg2*Δ (red squares), *erg3*Δ (green triangles), *erg4*Δ (yellow triangles) and *erg6*Δ (cyan hexagons) cells after addition of 10 μM CCCP (full symbols); control (empty symbols). (C) Changes in diS-C_3_(3) staining of wild-type cells (grey and black symbols) after addition of CCCP or FK506: 5 μM CCCP (diamonds), 10 μM CCCP (squares), 20 μM CCCP (inverted triangles), 10 μM FK506 (hexagons), 100 μM FK506 (triangles), control (circles) in comparison with staining of *pdr5*Δ (pink symbols) and *snq2*Δ cells (violet symbols): 10 μM CCCP (squares), 10 μM FK506 (hexagons), control (circles). (D) Changes in diS-C_3_(3) staining of wild-type (black circles), *erg2*Δ (red squares), *erg3*Δ (green triangles), *erg4*Δ (yellow triangles), *erg5*Δ (blue diamonds), *erg6*Δ (cyan hexagons), *pdr5*Δ (pink squares) and *snq2*Δ (violet hexagons) cells after addition of 100 μM FK506 (full symbols); control (empty symbols). Arrows with dotted lines indicate addition of compounds. (E) Growth inhibition zones of cells exposed to 30 mM FK506, 6.5 mM fluconazole, 5 mM NQO and their combinations.

To investigate how changes in sterol content influenced the ability of Pdr5p and also Snq2p to export drugs from cells, we performed a series of experiments. As mentioned above, the diS-C_3_(3) fluorescence probe is a substrate of both these pumps, as is CCCP (carbonyl cyanide 3-chlorophenylhydrazone) [31]. Thus in the presence of CCCP, the efflux of the probe via the Pdr5 and Snq2 pumps is inhibited (competitive inhibition between the two substrates) and the cell staining increases. In our experiments, CCCP was only able to effectively block the export of the probe from wild-type, *erg5*Δ, *pdr5*Δ and *snq2*Δ cells (Fig 5A), which suggested that at least one of the two MDR pumps was fully functional in these cells. We did not observe any similar increase in λ_max_ after the addition of 10 μM CCCP to *erg4*Δ, *erg6*Δ, *erg2*Δ and *erg3*Δ cells (Fig 5B) which indicated a broken activity of the MDR pumps in these strains. As the deletion of the *ERG4*, *ERG6*, *ERG2* and *ERG3* genes significantly altered the cell’s ability to export the fluorescence probe, which is a substrate of at least these two MDR pumps, we used several known substrates of these two pumps to determine whether Pdr5p and Snq2p were influenced by changes in the membrane composition in the same way. Whereas CCCP, fluconazole and the diS-C_3_(3) probe are substrates of both the Snq2 and Pdr5 pumps [31,37,45], the immunosuppressant FK506 is a specific substrate of Pdr5p [46] and the mutagen 4-nitroquinoline *N-*oxide (NQO) is a specific substrate of Snq2p [47].

First we compared the inhibition of the probe efflux by the addition of CCCP or FK506 in the wild-type cells and in the *pdr5*Δ and *snq2*Δ mutants. While 10 μM CCCP was sufficient for the inhibition of the probe export, as is evident from a concentration-dependent increase in λ_max_ after the exposure of cells to CCCP, even a tenfold higher concentration of FK506 was not able to cause a similar effect (Fig 5C). This result confirmed that CCCP effectively inhibited the probe export via both MDR pumps, as is also visible from the staining curves of *pdr5*Δ and *snq2*Δ cells exposed to CCCP. On the other hand, FK506 only affected Pdr5p (see Fig 5C; a small increase of diS-C_3_(3) staining of *snq2*Δ cells after addition of 10 μM FK506). The activity of Snq2 pump was unchanged by this drug (documented in Fig 5C by no change in staining after FK506 addition to *pdr5*Δ cells), and it was fully able to compensate the FK506-blocked probe export via Pdr5p in the wild-type cells. A similar result, i.e. no effect of FK506 addition was also observed in all *erg* mutants (Fig 5D). This result indirectly suggested that Snq2p was fully functional, in contrast to Pdr5p. Although some studies minimized the importance of the sterol composition of plasma membrane for the proper functioning of MDR pumps [48], it is generally assumed that the members of this protein family require close contact with neighbour components of the membrane to engage in their regular transport activity, as was demonstrated for Pdr5p [49] or Pdr12p [3]. This close interaction between MDR transporters and the surrounding sterols is limited in *erg* mutants, which mainly accumulate bended sterols instead of flat ergosterol in their membranes. In other words, the presence of double bonds in the aliphatic tail determines the planarity of the sterol molecules might affect, together with sphingolipids [3], the efflux capabilities of MDR pumps (Fig 1).

To confirm the results obtained in fluorescence measurements, we performed disc diffusion tests (Fig 5E and S3 Fig) and assessments of relative growth in liquid media (S4 Fig) with fluconazole as a substrate of both Pdr5p and Snq2p, FK506 as a specific substrate of Pdr5p, and NQO as a specific substrate of Snq2p. Pdr5p did not exhibit the same activity in all strains, because an increase in the size or clarity of the growth inhibition zones was visible in the presence of FK506, fluconazole and their combination in the *erg2*Δ, *erg3*Δ, *erg4*Δ and *erg6*Δ mutants (indicated by black frames in Fig 5E and red frames in Fig 4; the statistical analysis of the diameters of growth inhibition zones is shown in S3 Fig) compared to the growth inhibition zones of the parental strain BY4741, *erg5*Δ and *snq2*Δ mutants. In contrast to Pdr5p, Snq2p seemed to be fully functional in all strains, because the same size of zones was observed in the presence of NQO and its combination with fluconazole in all *erg* mutants and the wild type (white ellipses in Fig 5E). The results obtained by disc diffusion tests were confirmed by measurement of relative growth in liquid media. Obtained data are summarized in S4 Fig. Both approaches highlighted the significantly reduced activity of Pdr5p in *erg6*Δ and *erg4*Δ mutants. Since it is thought that Pdr5p is localized to ergosterol-rich domains of the plasma membrane [21], changes in the ergosterol content of the plasma membrane as a consequence of the deletion of genes involved in ergosterol biosynthesis have a significant influence on its function, whereas Snq2p seems to be less sensitive to changes in its microenvironment (Fig 5). Similarly, MDR transporters exhibit different lipid affinities in the pathogenic yeast *C*. *albicans*, where the inability to synthesize ergosterol leads to a mislocalization of Cdr1p and affects its functionality, whereas another MDR transporter, Mdr1p, remains functional and properly localized within the plasma membrane [50].

**Fig 6:**
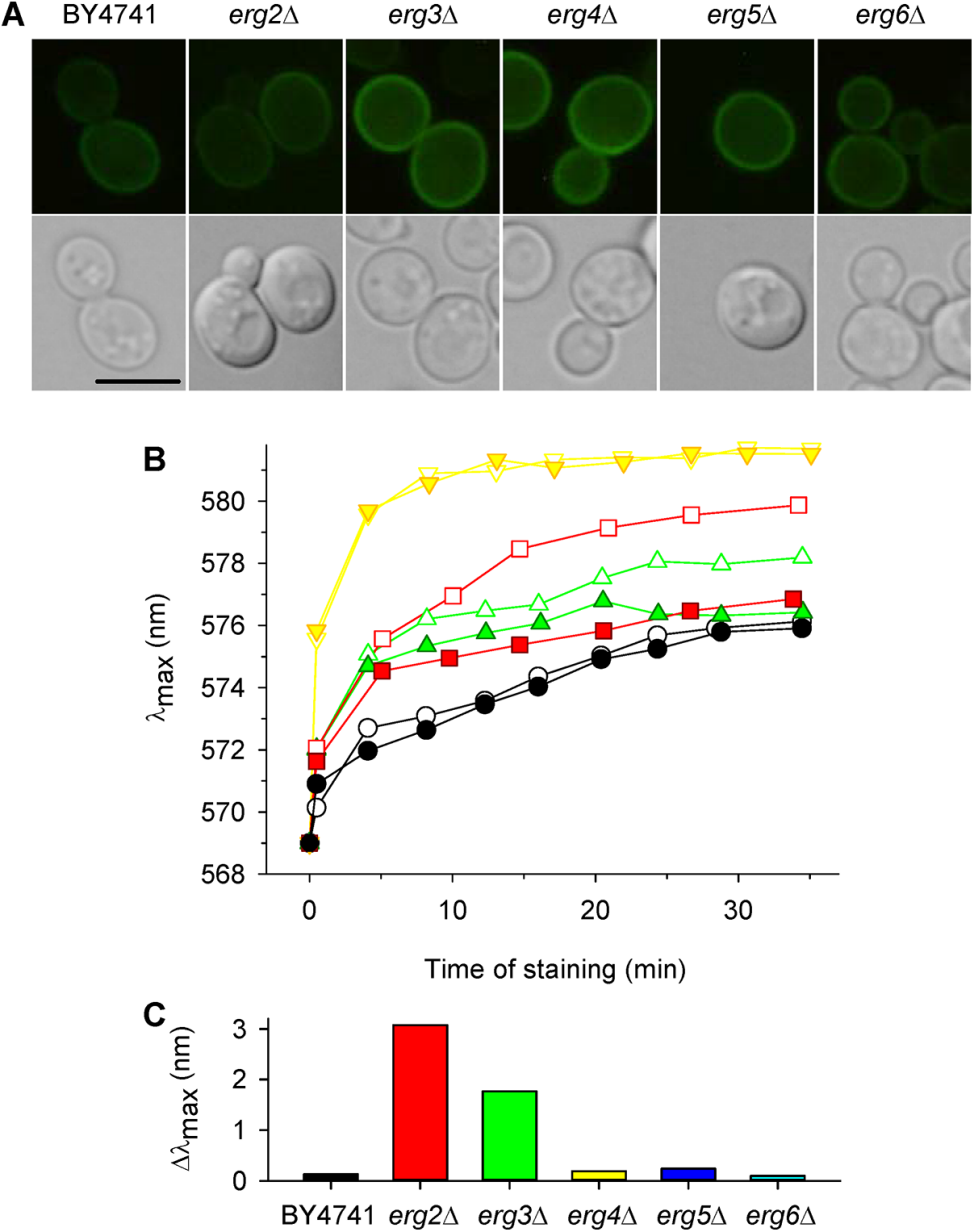
Overexpression of *PDR5*. (A) Deletion of *ERG* genes does not affect localization of Pdr5p fused with GFP (expressed from pGRUPDR5). The scale bar is 5 μm. (B) diS-C_3_(3) staining of wild-type BY4741 (black circles), *erg2*Δ (red squares), *erg3*Δ (green triangles) and *erg4*Δ (yellow triangles) cells transformed either with YEpPDR5 (overexpression of Pdr5p; full symbols) or YEp352 (empty plasmid; empty symbols). (C) Difference of Δλ_max_ between staining curves of cells transformed with YEp352 (empty plasmid) and YEpPDR5 illustrates an increase in the probe export caused by the Pdr5 overexpression. Values were calculated from panel B after 30 min of staining.

We were interested whether the lower activity of Pdr5p observed in several *erg* mutants resulted from a mislocalization and lower content of this transporter in their plasma membranes. To elucidate this possibility we constructed plasmids enabling overproduction and visualization (GFP-tagging) of Pdr5p. Then, the production and subcellular localization of Pdr5p were observed by monitoring the fluorescence emitted by the fused GFP in the wild type and *erg* mutants (Fig 6A). Green ring-shaped fluorescence at the cell surface was detected in all strains containing the pGRUPDR5 plasmid. The results indicated successful overproduction and correct localization of Pdr5p. Though deletion of *ERG* genes did not affect the localization of Pdr5p, the *PDR*5 overexpression did not improve significantly the cell capacity to export the fluorescence probe in all strains (an example is shown in Fig 6B). Only in the case of the *erg2*Δ and *erg3*Δ strains, Pdr5p overproduction improved slightly the capacity to export the probe (diminished level of diS-C_3_(3) staining of *erg2*Δ and *erg3*Δ cells overexpressing Pdr5p, Fig 6B and 6C). Both Erg2p and Erg3p change the B-ring of sterol molecule whereas other enzymes (Erg6, Erg5 and Erg4) modify the side chain of the sterol molecule as shown in Fig 1. This experiment showed that in spite of the correct localization of Pdr5p in *erg* mutants, its activity is lower than in the wild type, and the Pdr5p overexpression can improve the capacity to export the fluorescence probe only in *erg2*Δ and *erg3*Δ cells.

In summary, it is evident that the much higher diS-C_3_(3) staining of *erg2*Δ, *erg3*Δ, *erg4*Δ and *erg6*Δ mutants observed in the initial experiments (Fig 2A), was due to a combination of two effects–a hyperpolarization of the plasma membrane, and a lower activity of Pdr5p.

We found that the mutants, lacking genes encoding the final steps of ergosterol biosynthesis, differ in a number of parameters. Besides hyperpolarization of their membranes and a connected higher sensitivity to toxic alkali-metal cations and cationic drugs, we also observed differences in their response to high osmotic pressure and the ability to maintain a favourable intracellular pH upon osmotic-stress conditions. Moreover, we obtained a strong indication that the activity of one of the main MDR pumps in *S*. *cerevisiae*, Pdr5p, is strongly affected by the changed sterol composition of the plasma membrane.

In almost all the performed experiments, the phenotypes of the *erg4*Δ and *erg6*Δ mutants were the most different from the parental-strain phenotypes, suggesting that the accumulation of ergosterol precursors ergosta-5,7,22,24(28)-tetraenol and zymosterol, respectively, changes the properties of *S*. *cerevisiae* plasma membrane more significantly than the accumulation of other precursors. Thus *ERG6* and *ERG4* genes and their protein products may become useful antifungal targets for a new generation of inhibitors of ergosterol biosynthesis. *ERG6* is especially interesting, because it encodes sterol C-24 methyltransferase, which is not involved in cholesterol biosynthesis in mammalian cells. The deletion of the *ERG6* gene is neither lethal in *S*. *cerevisiae* nor in *C*. *albicans* [51], nevertheless it results in a series of serious effects. Besides those shown in our study, they include e.g. the inability to import tryptophan and utilize respiratory energy sources, and a hypersensitivity to a number of metabolic inhibitors [16,51–52]. The administration of inhibitors of Erg6p would make the pathogenic yeast hypersensitive to currently known and established antifungal agents as well as to new compounds. Because of the increased drug access produced by inhibitors of the sterol C-24 methyltransferase, other antifungal drugs will become more effective and can be clinically applied at reduced dosages, limiting their possible negative side effects. Though some promising compounds with *in vitro* activities against Erg6p have already been synthesised (e.g. [53–54]), much more work is still needed to develop new and efficient antifungal drugs targeting ergosterol biosynthesis, mainly due to a rapidly increasing number of clinical isolates resistant to classical ergosterol-pathway-targeted antifungal drugs [55–56].

## Supporting Information

S1 Fig Growth of *Saccharomyces cerevisiae* wild-type BY4741 and isogenic *erg* mutant strains on solid media. Tenfold serial dilutions of saturated cultures were prepared and 3-μL aliquots spotted onto YPD or YNB plates and incubated at 30°C. The *erg6*Δ strain is a new construct, see Table 1 and Materials and methods.
(TIF)

S2 Fig Relative growth in liquid YPD medium supplemented with antifungal drugs. Cells were cultivated in the presence of (A) 2 nM cycloheximide, (B) 1 μM nystatin, (C) 0.1 μM clotrimazole, (D) 1 μM ketoconazole, (E) 5 μM fluconazole and (F) 0.2 μM itraconazole. Growth without drugs = 100%. The *P* values (*P < 0.05, **P < 0.01, ***P < 0.001) denote statistically significant differences from the wild-type strain.
(TIF)

S3 Fig Diameters of the growth inhibition zones. The cells were exposed to (A) 30 mM FK506, (B) 6.5 mM fluconazole, (C) 5 mM NQO and their combinations (D) 6.5 mM fluconazole plus 30 mM FK506, (E) 6.5 mM fluconazole plus 5 mM NQO. Diameter of paper discs was 6 mm. The *P* values (*P < 0.05, **P < 0.01, ***P < 0.001) denote statistically significant differences from the wild-type strain.
(TIF)

S4 Fig Relative growth in liquid YPD medium supplemented with antifungal drugs. Cells were cultivated in the presence of (A) 10 μM fluconazole, (B) 20 μM FK506, (C) 0.1 μM NQO and their combinations (D) 10 μM fluconazole plus 20 μM FK506 and (E) 10 μM fluconazole plus 0.1 μM NQO. Growth without drugs = 100%. The *P* values (*P < 0.05, **P < 0.01, ***P < 0.001) denote statistically significant differences from the wild-type strain.
(TIF)

S1 Table Oligonucleotides used in this study. (DOC)
