# Supplementary figures and images for: Changes in the Sterol Composition of the Plasma Membrane Affect Membrane Potential, Salt Tolerance and the Activity of Multidrug Resistance Pumps in Saccharomyces cerevisiae
Source: PLoS One. 2015 Sep 29;10(9):e0139306. doi: 10.1371/journal.pone.0139306 (PMC4587746; doi:10.1371/journal.pone.0139306)

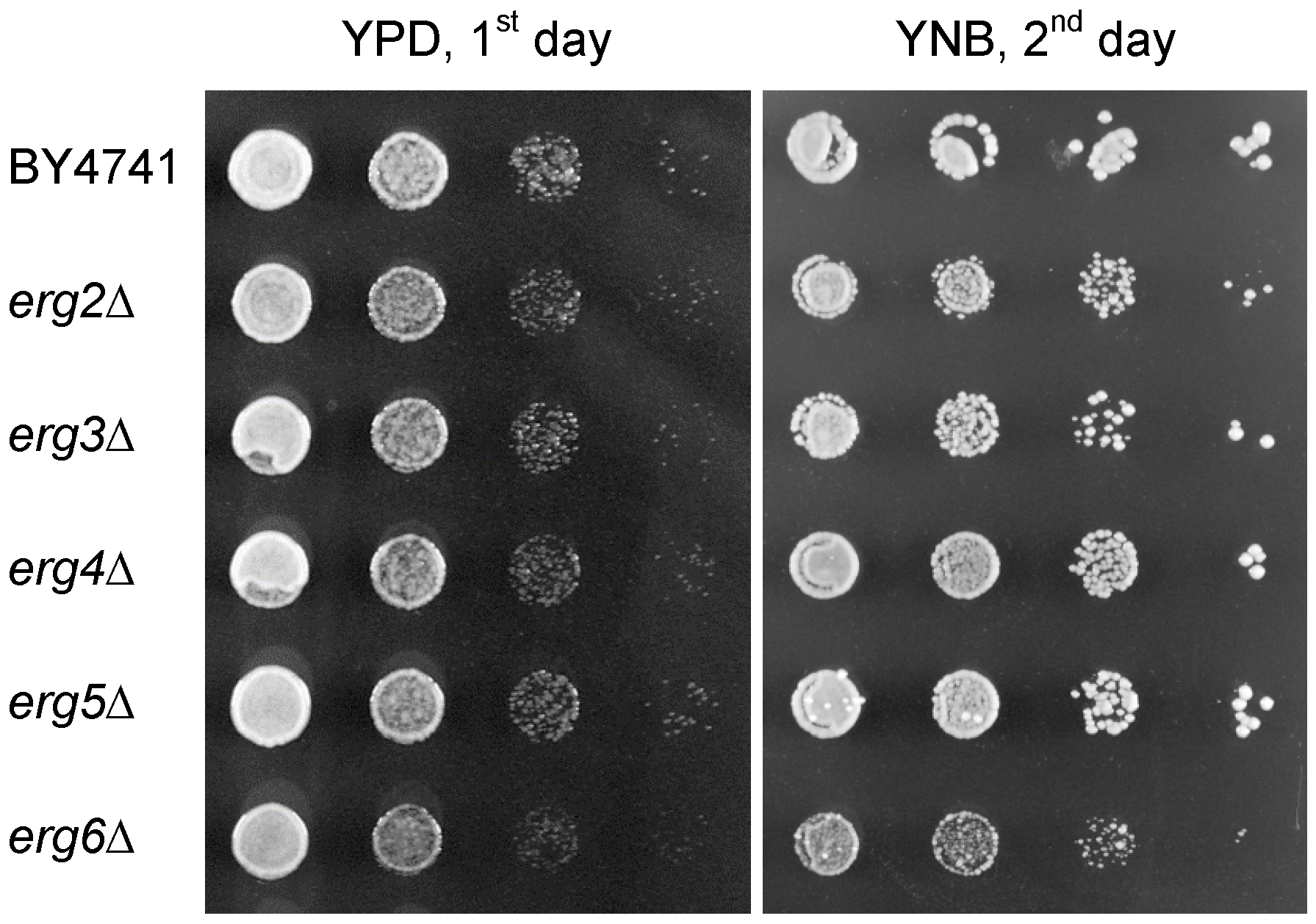

Supplement: S1 Fig — Tenfold serial dilutions of saturated cultures were prepared and 3-μL aliquots spotted onto YPD or YNB plates and incubated at 30°C. The erg6Δ strain is a new construct, see Table 1 and Materials and methods. (TIF) [file pone.0139306.s001.tif]

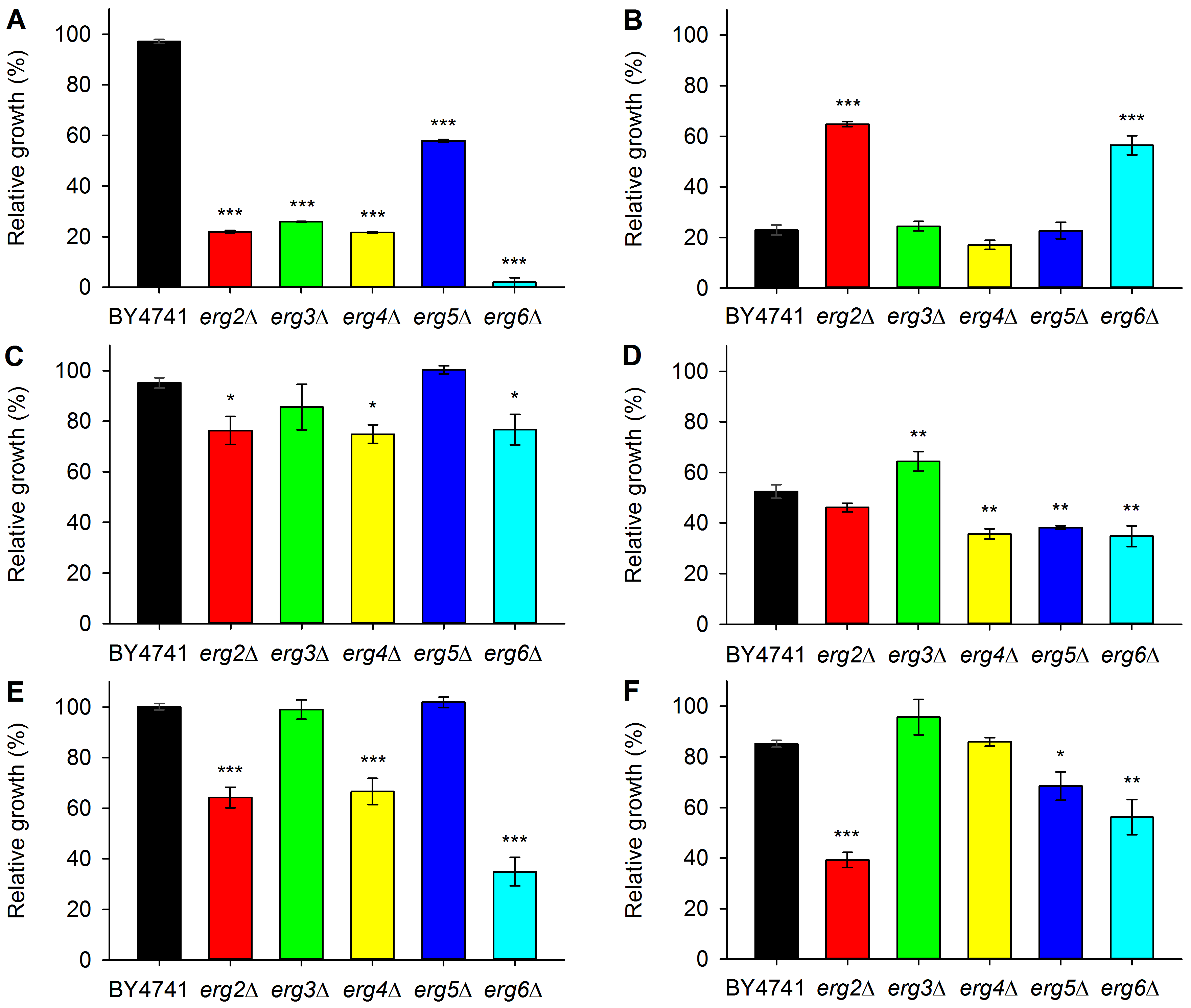

Supplement: S2 Fig — Cells were cultivated in the presence of (A) 2 nM cycloheximide, (B) 1 μM nystatin, (C) 0.1 μM clotrimazole, (D) 1 μM ketoconazole, (E) 5 μM fluconazole and (F) 0.2 μM itraconazole. Growth without drugs = 100%. The P values (*P < 0.05, **P < 0.01, ***P < 0.001) denote statistically significant differences from the wild-type strain. (TIF) [file pone.0139306.s002.tif]

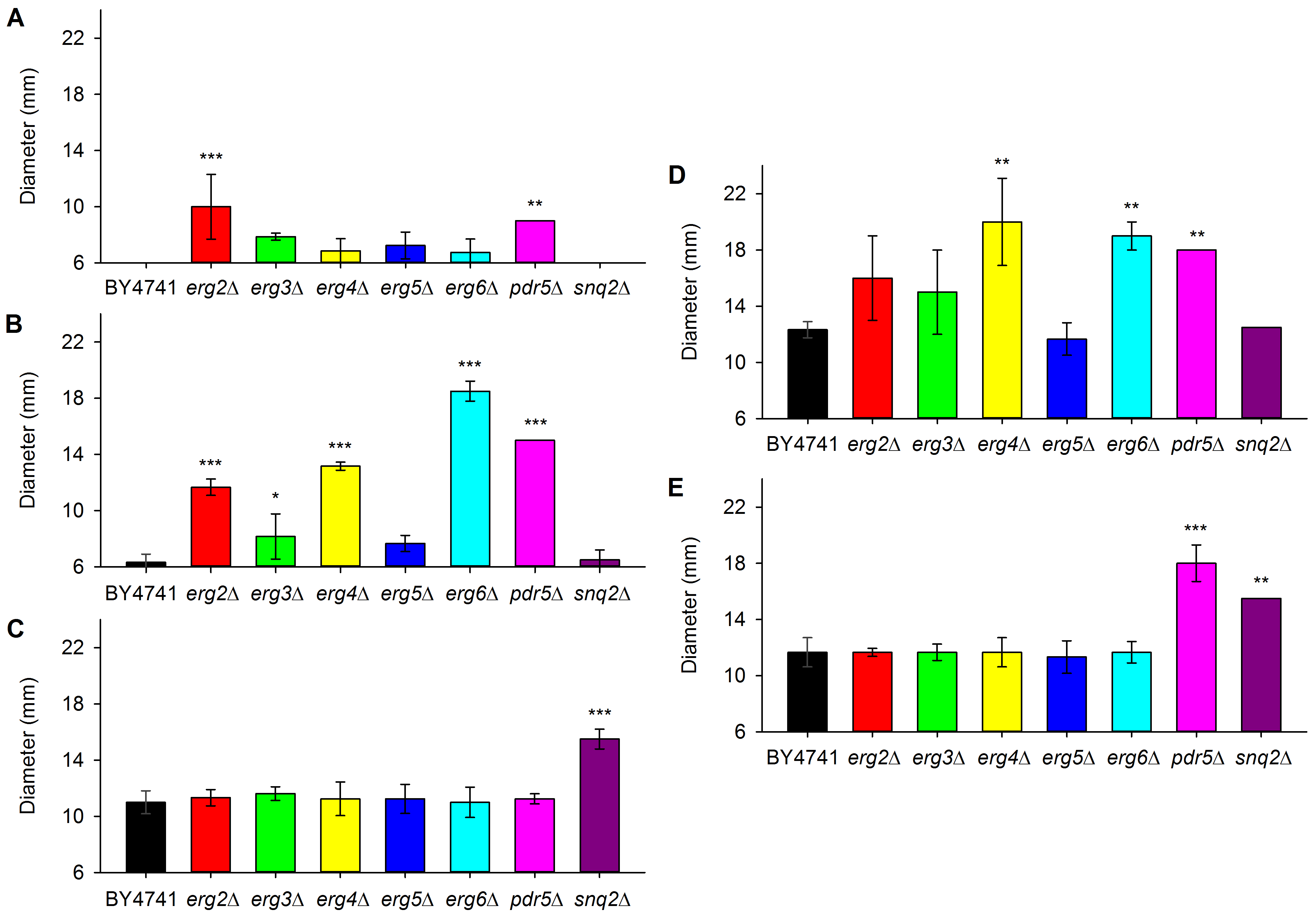

Supplement: S3 Fig — The cells were exposed to (A) 30 mM FK506, (B) 6.5 mM fluconazole, (C) 5 mM NQO and their combinations (D) 6.5 mM fluconazole plus 30 mM FK506, (E) 6.5 mM fluconazole plus 5 mM NQO. Diameter of paper discs was 6 mm. The P values (*P < 0.05, **P < 0.01, ***P < 0.001) denote statistically significant differences from the wild-type strain. (TIF) [file pone.0139306.s003.tif]

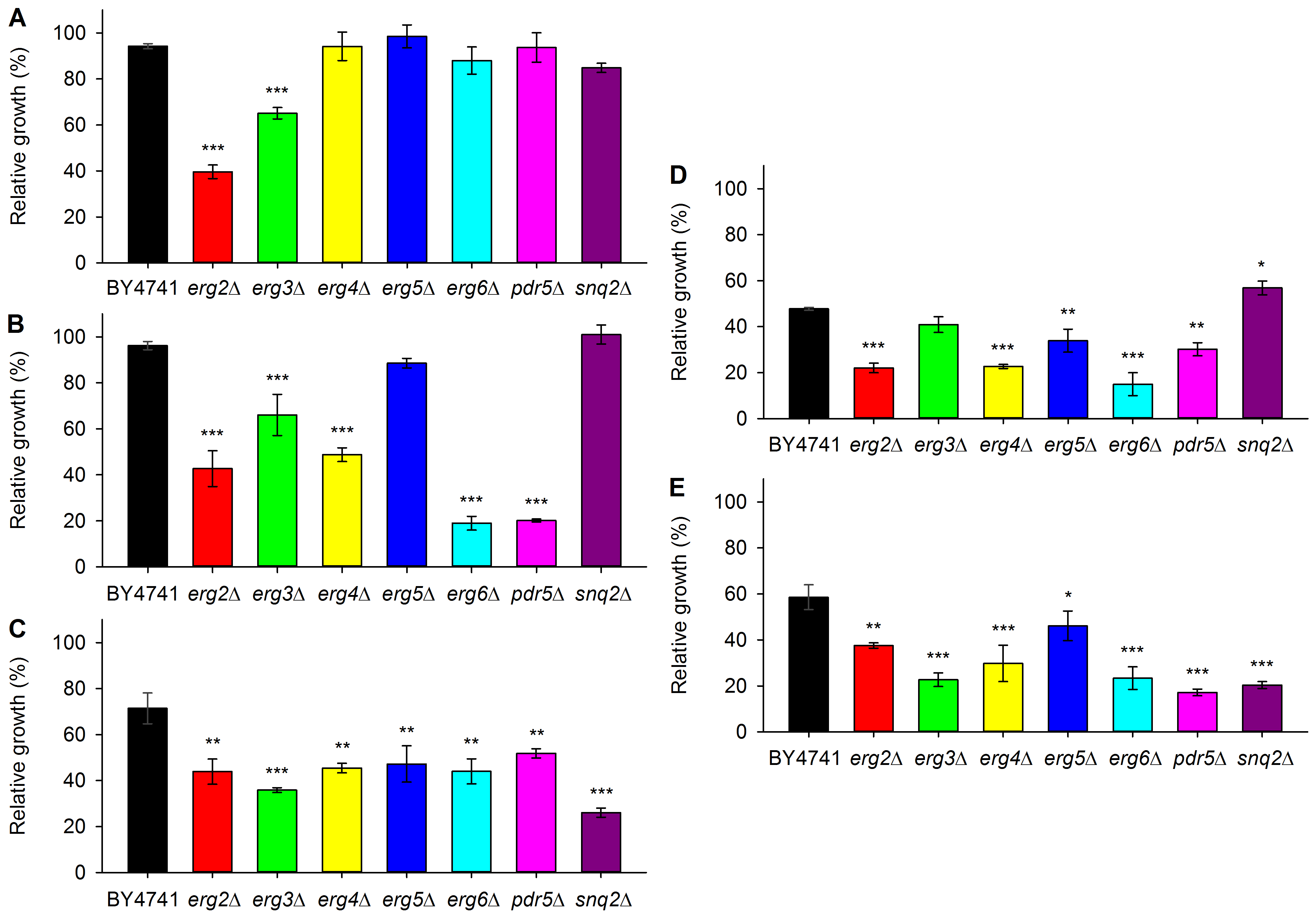

Supplement: S4 Fig — Cells were cultivated in the presence of (A) 10 μM fluconazole, (B) 20 μM FK506, (C) 0.1 μM NQO and their combinations (D) 10 μM fluconazole plus 20 μM FK506 and (E) 10 μM fluconazole plus 0.1 μM NQO. Growth without drugs = 100%. The P values (*P < 0.05, **P < 0.01, ***P < 0.001) denote statistically significant differences from the wild-type strain. (TIF) [file pone.0139306.s004.tif]
