# Supplementary material for: Changes in the Sterol Composition of the Plasma Membrane Affect Membrane Potential, Salt Tolerance and the Activity of Multidrug Resistance Pumps in Saccharomyces cerevisiae
Source: PLoS One. 2015 Sep 29;10(9):e0139306. doi: 10.1371/journal.pone.0139306 (PMC4587746; doi:10.1371/journal.pone.0139306)
Supplement: S1 Table — (DOC) [file pone.0139306.s005.doc]

**S1 Table. Oligonucleotides used in this study**

| **Name** | **Sequence** |
| --- | --- |
| ScERG6-KanMX-F | caagaataaaataataatatagtaggcagcataagatgagttcgtacgctgcaggtcgac |
| ScERG6-KanMX-R | gtatatatcgtgcgctttatttgaatcttattgatctagtgagcataggccactagtggatctg |
| ScERG6-UP-F1 | gatgcaacagggtaagatcag |
| ScERG6-R2 | gtgtggcttcattataatcc |
| ScERG6-F2 | ctacaaggttttgaaaccgg |
| ScERG6-D-R1 | ggcctgctagcaatgaacgtg |
| PDR5-YEp-F | gtacattataaaaaaaaatcctgaacttagctagatattatgcccgaggccaagcttaac |
| PDR5-YEp-R | cacgacgttgtaaaacgacggccagtgccaagcttgcatgttatttcttggagagtttaccg |
| PDR5-pGRU-R | taaagctccggagcttgcatgcctgcaggtcgactcttttcttggagagtttaccgttc |
| ScPDR5-1R | cgggtttacaccttccacac |
| ScPDR5-2F | gccactgacacctgtagt |
| NHA1-1-F | gccaactctgtgtgatatagc |
| YEp352-R | ggggatgtgctgcaaggcg |
| RGFP-1 | ctgggtatctcgcaaaac |
